# Association between Salivary Hormones, Dental Caries, and Cariogenic Microorganisms during Pregnancy

**DOI:** 10.3390/jcm13113183

**Published:** 2024-05-29

**Authors:** Ruqian Yang, Xingyi Lu, Nora Alomeir, Sally Quataert, Tongtong Wu, Jin Xiao

**Affiliations:** 1 Eastman Institute for Oral Health, University of Rochester Medical Center, Rochester, NY 14620, USA; ruqian_yang@urmc.rochester.edu (R.Y.); nora_alomeir@urmc.rochester.edu (N.A.); 2 Biostatistics and Computational Biology, University of Rochester Medical Center, Rochester, NY 14620, USA; xingyi_lu@urmc.rochester.edu (X.L.); tongtong_wu@urmc.rochester.edu (T.W.); 3 Immunology and Microbiology, University of Rochester Medical Center, Rochester, NY 14620, USA; sally_quataert@urmc.rochester.edu

**Keywords:** caries, *Streptococcus mutans*, *Candida albicans*, hormone, pregnancy

## Abstract

**Objective**: This study aimed to identify the salivary levels of six hormones (progesterone, estradiol, testosterone, cortisol, thyroxine T3, and triiodothyronine T4) in pregnant women, and to assess the association between salivary hormones, dental caries, and cariogenic microorganisms. **Methods**: This cross-sectional study included 181 low-income US pregnant women who were in their third trimester. Demographic details, oral hygiene practices, and medical backgrounds were obtained via questionnaires and medical records. Calibrated dentists obtained data on plaque index and caries status through comprehensive oral examinations. Unstimulated saliva was collected 2 h before eating and brushing. Salivary hormones were measured with a multiplex assay. Oral *Streptococcus mutans* (*S. mutans*) and *Candida albicans* (*C. albicans*) were quantified via colony-forming unit (CFU) counts. A latent model was used to generate clusters of pregnant women based on salivary hormone levels, followed by post-clustering analysis. Factors associated with salivary cariogenic microorganisms were further evaluated via multiple regression analyses. **Results**: Estradiol, progesterone, testosterone, cortisol, T3, and T4 in saliva were detectable at rates of 92%, 97%, 77%, 99%, 71%, and 50%, respectively. Three distinct participant clusters (high, intermediate, and low) were identified based on salivary hormone levels. Intermediate-level and high-level clusters had increased numbers of decayed teeth, decayed surfaces, ICDAS scores, and salivary *S. mutans* and *C. albicans*, compared to the low-level cluster (*p* < 0.05). Covariate analysis demonstrated that the high-level cluster was positively associated with salivary carriage of *S. mutans* (CFU/mL) (*p* < 0.05). Participants with higher levels of progesterone, estradiol, testosterone, and cortisol were associated with a high carriage status of *S. mutans* in saliva (>10^5^ CFU/mL) (*p* < 0.05). **Conclusions**: This study demonstrated the feasibility of detecting salivary hormones during pregnancy and revealed the positive association between salivary steroid hormones and cariogenic pathogens.

## 1. Introduction

Women experience a greater incidence of untreated dental caries compared to men [1]. It has been observed that dental caries occur 1.97 times more frequently in pregnant women than in non-pregnant women [2]. Up to 87.2% of pregnant women have dental caries and pregnant women in families below the federal poverty level (FPL) have a higher prevalence of untreated dental caries compared to non-poor families above FPL [3]. Pregnancy is marked by significant fluctuations of various hormones, such as sex steroids, cortisol, and thyroid hormones [4,5]. Hormonal changes during pregnancy increase host susceptibility to oral infectious diseases such as gum diseases and dental caries, affecting not only expectant mothers but also developing fetuses [6]. However, our current knowledge about the relationship between hormones and dental caries during pregnancy remains unclear.

Throughout pregnancy, alterations in the quantity and diversity of microbiomes have been noted in the gut, oral cavity, and vagina [7]. In the oral cavity, a few studies have indicated potential associations between sex hormone levels, host immune suppression, and oral microbiota shifts, which may lead to a higher prevalence of oral diseases [8]. Despite research in this field being limited, the evidence available suggests that changes in sex steroids during pregnancy may influence the balance between the host and its microbes in the oral cavity [9]. Fluctuations in estrogen and progesterone levels during pregnancy have been reported to cause dilatation and tortuosity of gingival microvasculature, circulatory stasis, and increased oral vasculature permeability. Previous studies have also suggested that female sex steroids play a role in selecting black-pigmented *Bacteroides*, especially the periodontal pathogen, *Prevotella intermedia* [10]. As reported, augmented salivary sex hormonal levels are associated with an increased proportion of anaerobic and aerobic bacteria, such as *Bacteroides melaninogenicus*, *Bifidobacterium dentium*, *Prevotella intermedia*, and *Porphyromonas gingivalis* [11,12]. Furthermore, studies showed a higher detection of *Candida* spp. among women in their late pregnancy stage, compared to the non-pregnant group [13,14].

*Streptococcus mutans* has traditionally been considered the primary microbial risk marker for dental caries [15,16,17]. A meta-analysis reported an increase in the salivary *S. mutans* count during the second and third trimesters, although the contributing factors behind this phenomenon remain to be explored [18]. Recently, oral *Candida* spp. carriage, especially *Candida albicans*, has been reported to be positively associated with the number of decayed teeth during pregnancy [15,17]. Interestingly, a higher detection of *Candida* spp. among women was exclusively found in the late stage of pregnancy compared to the non-pregnant group [13,14]. It is known that key hormones like estradiol, progesterone, and cortisol peak in the third trimester [4], while the relationship between salivary hormones, dental caries status, and cariogenic pathogens during pregnancy has not been fully elucidated. Although some work has been conducted to explore the correlation between salivary hormones and oral microbiota, previous studies mainly focused on individual hormones and the overall pattern of multiple hormones in saliva during pregnancy has not been studied [19,20].

Given the above, the aim of this study is to identify the levels of six hormones (progesterone, estradiol, testosterone, cortisol, thyroxine T3, and triiodothyronine T4) in the saliva of underserved pregnant women, to assess the clusters of the six salivary hormones, and to explore the association between salivary hormones, dental caries, and cariogenic pathogens.

## 2. Materials and Methods

### 2.1. Study Population and Design

The data and samples from this study were obtained from the parent cohort study involving 186 pregnant women, who were selected from the patient demographic of economically disadvantaged women attending the University of Rochester Medical Center (URMC) Highland Family Medicine (HFM) and Eastman Institute for Oral Health (EIOH) between 2017 and 2020 [21,22,23]. Out of the initial 186 participants enrolled, 181 completed the collection of saliva samples and were included in the present study. The socioeconomic status of the study participants was determined based on their New York state-supported Medicaid insurance type, which reflected their income levels (≤138% of the FPL) and rendered them eligible for participation. Written informed consent was obtained from all participants. The study protocol received approval from the University of Rochester Research Subject Review Board (no. 1248).

### 2.2. Inclusion and Exclusion Criteria

All individuals who met the specified inclusion and exclusion criteria were included in the study. The inclusion criteria consisted of (1) being over 18 years of age, (2) being beyond 28 weeks of gestation, and (3) being pregnant with a single fetus. Conversely, the exclusion criteria included consisted of (1) having severe systemic diseases, such as acquired immunodeficiency syndrome (AIDS), making them more vulnerable to infections, (2) being diagnosed with oral cancer, (3) having a maxillofacial deformity, such as a cleft lip/palate, and (4) using antifungal medication 3 months before the study enrollment.

### 2.3. Data Collection and Examination

Comprehensive oral examinations were performed by one of three calibrated dentists using standard equipment. Dental plaque was assessed utilizing the Plaque Index (PI) method developed by Löe and Sillnes [24], where each of the four gingival areas of the tooth received a score ranging from 0 to 3. Dental caries was evaluated using the WHO standard of DMFT (decayed, missing, and filled teeth) index and the International Caries Detection and Assessment System (ICDAS). The inter- and intra-examiner agreements for the evaluated criteria were assessed using Kappa statistics, demonstrating a consistency exceeding 83% during the calibration process. Saliva sample collection was detailed previously [21]. Prior to the sample collection, study subjects were instructed to abstain from eating, drinking, or brushing their teeth for 2 h. Approximately 3–5 mL of unstimulated whole saliva samples were collected by participants spitting into a sterile 50 mL centrifuge tube. All samples were transported to the lab within 2 h of collection and stored in a −80 °C freezer [24]. Demographic and socioeconomic data (age, race, ethnicity, educational level, marital status, and employment status) were collected using a self-reported questionnaire. Participants also provided self-reported data on oral health behaviors, including smoking status and brushing habits. Medical data were acquired through self-reporting and confirmation using URMC electronic health records (eRecord), encompassing physician-diagnosed medical conditions such as diabetes, asthma, hypertension, and any recent history of yeast infection.

### 2.4. Identification and Quantification of S. mutans and C. albicans

The clinical samples were stored on ice and transferred to the Center for Oral Biology, University of Rochester (within 2 h) for laboratory testing. Upon arrival, the saliva samples were gently vortexed and sonicated to break down any aggregates before plating. The sonication process involved repeating three cycles, each consisting of 10 s of sonication followed by 30 s of rest on ice. BBL™ CHROMagar™ Candida (BD, Sparks, MD, USA) was used for isolating *Candida* spp., and Mitis Salivarius with Bacitracin selective medium was used for *S. mutans*. The plates were incubated at 37 °C, 5% CO_2_, for 48 h before identification. For further identification of species that could not be discerned by colony morphology, Colony PCR was employed [21]. The colony-forming unit (CFU) values of *S. mutans* and *C. albicans* on each plate were recorded.

### 2.5. Salivary Hormone Measurement

The assessment of six hormonal analytes was conducted using a multiplex assay. Salivary samples were centrifuged for 5 min at 125,000 relative centrifugal force (RCF) at 4 °C prior to overnight incubation at 4 °C. Assays were carried out following the manufacturer’s instructions for the MILLIPLEX MAP Multi-Species Hormone Magnetic Bead Panel (Cat no. MSHMAG-21K). The results were obtained using a Luminex 200 instrument and reported based on standard curve values.

### 2.6. Statistical Analysis

All statistical tests were conducted with a two-sided significance level of 5%. Statistical analyses were performed using R version 4.2.2 (R Foundation for Statistical Computing, Vienna, Austria). CFU values were converted to natural log values, and zero values remained unchanged. The Shapiro–Wilk test, Q-Q plot, and density plot were used to examine whether the datasets were normally distributed. Transformations were employed for data exhibiting non-normal distribution.

Latent modeling and the K-means method were used for clustering. The comparisons of continuous variables between groups were conducted using *t*-tests and one-way ANOVA, while chi-square tests were conducted in the analyses of contingency tables. Multinomial logistic regression was used to evaluate the impact of independent variables on the classification of patients.

## 3. Results

Pregnant women recruited in this study were patients visiting the University of Rochester Highland Family Medicine (HFM) or Eastman Institute for Oral Health (EIOH) Perinatal Dental Clinic. Both clinics, HFM and EIOH, serve predominantly low-income patients. Overall, among 181 subjects who completed the study, 53% were Black, 29.3% were White, and 17.7% consisted of other racial backgrounds. The subjects had an average age of 28 years old, and they were, on average, 33 weeks into their gestation.

### 3.1. Hormonal Levels and Clusters of Saliva during the Third Trimester

**Figure 1:**
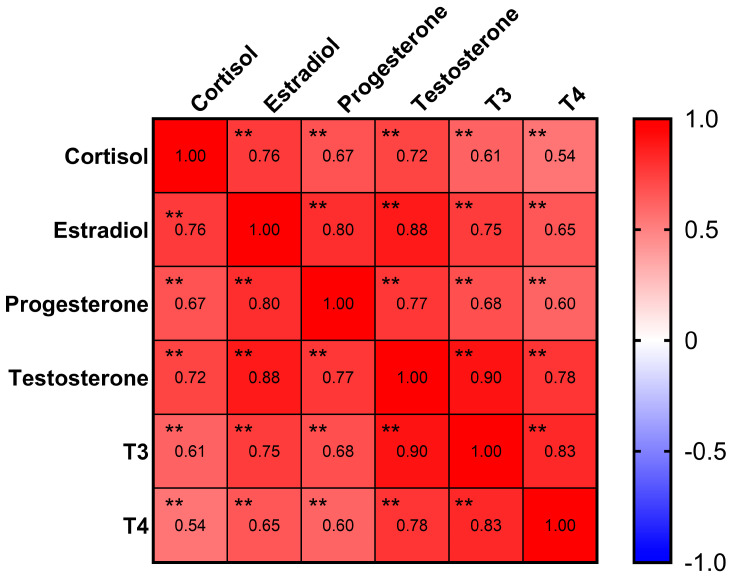
Correlation matrix of hormones. Progesterone, estradiol, and testosterone had strong correlations in between. ** *p* < 0.01.

Estradiol, progesterone, testosterone, cortisol, thyroxine (T3), and triiodothyronine (T4) were detectable with rates of 92%, 97%, 77%, 99%, 71%, and 50%, respectively (Table 1). The Pearson correlation analysis (Figure 1) indicated strong correlations (coefficient > 0.6) between all six hormones (*p* < 0.01). Notably, an even stronger correlation with a coefficient > 0.8 was found between the following sex hormones: estradiol, progesterone, and testosterone (*p* < 0.01).

**Figure 2:**
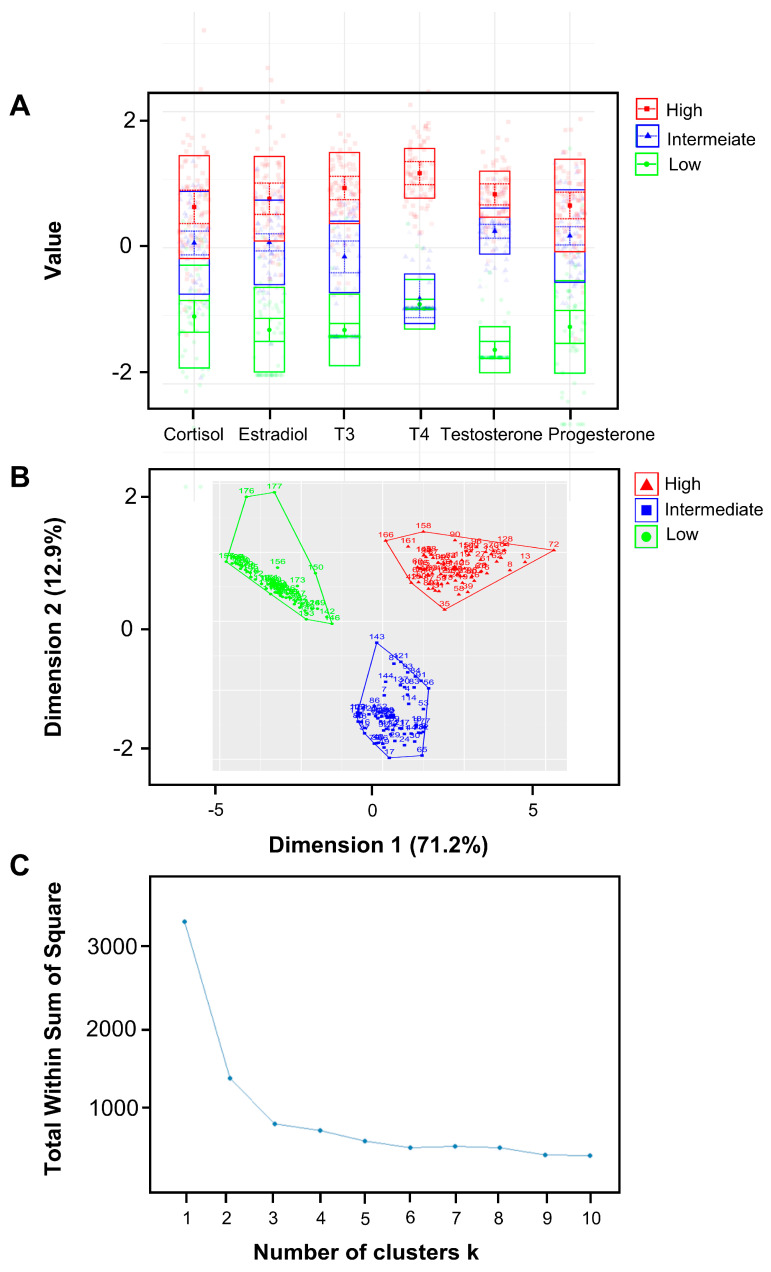
Clusters of pregnant women based on hormone level patterns with latent profile analysis (LPA). (**A**) A latent model was used to generate three clusters of pregnant women based on their salivary hormone levels. Three clusters were distinguished from 181 samples, including the clusters of low hormone levels, intermediate hormone levels, and high hormone levels. There is minimal overlap among the three classes, signifying that the classification effectively differentiates based on hormone levels. (**B**,**C**) The K-means clustering method yields results remarkably similar to those obtained using LPA, with 170 out of 181 subjects being classified into the same cluster by both methods. Additionally, the K-means method produces a flawless cluster plot for three clusters; the plot—indicating the optimal number of clusters—also suggests that three is the ideal number. Consequently, we selected three as the number of clusters.

The latent profile analysis (LPA) was utilized to generate clusters of pregnant women based on their salivary hormone levels. Three clusters were identified and characterized by low, intermediate, and high hormone levels (Figure 2). The K-means clustering method yielded results that closely resembled those obtained using LPA and further verified that the K-means method produced a cluster plot for three clusters without any anomalies, supporting three as the ideal number of clusters.

### 3.2. Demographic–Medical–Oral Characteristics of All Subjects and Three Hormonal Clusters

Demographic, medical, and oral health characteristics of the 181 pregnant women are shown in Table 2. Dental caries status—reflected by the decayed teeth (DT) and decayed surface (DS) numbers—was more significant in the higher levels of hormonal clusters and increased with the cluster level. Specifically, decayed surfaces in the low-level cluster were 2.7 ± 4.6 on average and increased to 3.5 ± 4.1 in the intermediate-level cluster and 5.5 ± 10.2 in the high-level cluster (*p* < 0.05). Increased carriage of the cariogenic pathogens *S. mutans* and *C. albicans* were also found in higher hormonal cluster levels (*p* < 0.05), with a significant difference between the low-level and intermediate-level clusters. Portions of Black pregnant women increased with higher cluster levels, while the opposite trend was found in White pregnant women. Moreover, higher portions of smokers were found in the intermediate (17.9%) and high (19.5%) level clusters, compared to 4.2% of smokers in the low-level cluster.

### 3.3. Factors Associated with Clusters of Salivary Hormones

According to the results from the univariate analysis mentioned above, variables of interest were selected, and logistic regression was applied to explore the factors potentially associated with the salivary hormone clusters during late pregnancy (Table 3). Results showed that *S. mutans* carriage was positively and significantly associated with the higher-level hormonal cluster when the high-level cluster was compared with the low-level cluster (*p* < 0.05). The plaque index (PI) was also positively associated with the higher-level clusters of salivary hormones when intermediate-level and high-level clusters were compared with the low-level cluster, respectively (*p* < 0.05). The Black race was found to be significantly related to the intermediate-level cluster when compared to the low-level cluster (*p* < 0.05), while no significance existed when comparing the high-level cluster with the low-level and intermediate-level clusters, respectively (*p* > 0.05).

### 3.4. Hormones Associated with S. mutans Carriage in Saliva

**Figure 3:**
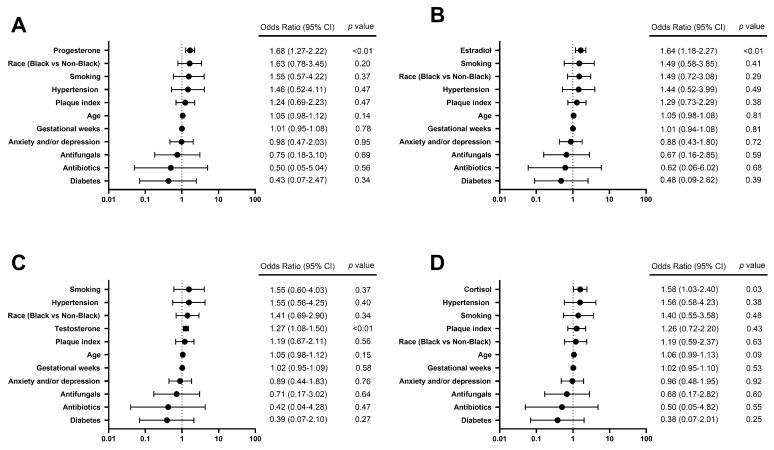
Forest plots of logistic regression models show factors associated with a high caries risk status indicated by salivary *S. mutans* ≥ 10^5^ CFU/mL. The odds of a high caries risk status of salivary *S. mutans* ≥ 10^5^ CFU/mL are predicted to (**A**) increase by 1.68 times with each additional unit of Ln (progesterone, pg/mL) (*p* < 0.01), (**B**) increase by 1.64 times with each additional unit of Ln (estradiol, pg/mL) (*p* < 0.01), (**C**) increase by 1.55 times with each additional unit of Ln (testosterone, pg/mL) (*p* < 0.01), and (**D**) increase by 1.58 times with each additional unit of Ln (cortisol, pg/mL) (*p* < 0.05).

To further explore the relationship between individual hormones of interest and *S. mutans* carriage in saliva, salivary *S. mutans* ≥ 10^5^ CFU/mL was chosen as a cutoff to reflect the individual status of high caries risk [15]. Among six hormones of interest, increased concentrations of estradiol, progesterone, testosterone, and cortisol were observed in the group of *S. mutans* ≥10^5^ CFU/mL, compared to the group of *S. mutans* <10^5^ CFU/mL (*p* < 0.05) (Table 4). Due to the strong correlations between each hormone, every single hormone of interest was analyzed with other variables of interest via regression analysis independently. The results (Figure 3) further revealed that salivary progesterone, estradiol, testosterone, and cortisol were positively associated with the status of *S. mutans* ≥10^5^ CFU/mL, with odds ratios of 1.68 (95% CI 1.27–2.22), 1.64 (95% CI 1.18–2.27), 1.55 (95% CI 1.08–1.50), and 1.58 (95% CI 1.03–2.40), respectively (*p* < 0.05).

## 4. Discussion

It has been reported that unstimulated saliva is optimal for determining female hormones [12]. The results from our study showed that the sex steroids progesterone, estradiol, testosterone, stress hormone cortisol, and the thyroid hormones T3 and T4 were detectable in saliva during the third trimester of pregnancy. Consistent with the previous studies, estradiol, progesterone, and cortisol in our study showed high detection rates of up to 99%, while testosterone had a lower detection rate of 77%. The significant rise in estradiol, progesterone, and cortisol during pregnancy is widely acknowledged and can be explained by the elevated activities of the corpus luteum, the maternal ovary, the adrenal cortex, and the placenta during pregnancy. Previous studies have reported that during pregnancy, the levels of estrogen and progesterone, either in serum or saliva, remarkably increase—up to 50 times more than during the non-pregnant period—and peak in the late trimester [25,26]. Longitudinal studies have also documented a weaker and gradual increase in testosterone concentration throughout gestation [27]. Similar to the change in sex steroids, the steroid hormone cortisol attains maximal levels during late pregnancy [4]. Meanwhile, an increase in thyroxine-binding globulin (TBG), thyroxine consumption by the fetus, and placental type 3 deiodinase expression require upregulation of thyroid hormone production to maintain adequate thyroid hormone availability [28,29]. With limited published data on T3 and T4 detection in saliva, our study confirmed that T3 and T4 were detectable in saliva during late pregnancy with the multiplex assay. However, a more sensitive approach is suggested for future studies due to the relatively low level of thyroid hormones in saliva.

The correlation analysis further revealed that the six hormones of interest were strongly correlated. Potential direct and indirect hormonal interactions have been reported before. One study revealed that amniotic fluid cortisol was a significant predictor of amniotic fluid testosterone [30]. Another study found that progesterone was able to compete with cortisol binding, therefore increasing the free cortisol fraction during pregnancy [31]. Changes in steroid hormones during pregnancy have also been found to affect thyroid hormones. It has been reported that thyroid-stimulating hormone (TSH) and human chorionic gonadotropin (hCG) have similar biomolecular structures and, therefore, hCG can cross-react with TSH, further stimulating the thyroid gland [5]. The strong correlation between hormones indicated that hormone patterns in saliva as a whole may act differently in the oral cavity than individual hormones do, which further intrigued us to study the salivary hormone clusters and to explore their relationship with dental caries conditions and cariogenic pathogens. In our study, three hormonal clusters were successfully identified via the latent model to represent the high, intermediate, and low levels of hormones in saliva. The minimal overlap among the three classes signified that the classification effectively differentiated based on hormone levels. Additionally, the K-means method further confirmed that three is the optimal number for clustering. This was evidenced by classification results nearly identical to those from LPA and that plot of the curve of the total within the sum of squares (WAS) identified three as the ideal number of clusters.

Regarding the potential factors associated with hormonal clusters, regression analysis—including potential covariates—demonstrated that being Black, having a high dental plaque index, and increased levels of the cariogenic pathogen *S. mutans* were significantly related to higher-level hormonal clusters compared to low-level clusters. Evidence from early studies showed that Black women had higher serum concentrations of testosterone and estradiol during pregnancy than White women or women of other races [32,33], and our study further confirmed the role of race relating to hormone levels in saliva.

Dental caries conditions have been found to be significantly associated with the hormones in saliva during late pregnancy. First, with an increased level of hormonal clusters, the numbers of both DT and DS, as well as ICDAS scores reflecting caries severity, significantly increased (*p* < 0.05). Meanwhile, cariogenic pathogens *S. mutans* and *C. albicans* in saliva also increased in intermediate-level and high-level clusters compared with low-level hormonal clusters. Covariate regression analysis further highlighted that higher-level clusters of salivary hormones were significantly associated with increased carriage of *S. mutans* in saliva (*p* < 0.05). A positive association was also presented between increased ICDAS and high hormone levels in saliva compared to the low-level cluster, although no significance was found. These results highlight the close relationship between salivary hormones, cariogenic pathogens, and dental caries status during late pregnancy. Commonly reported risk factors for dental caries included age, gender, socioeconomic status, race, geographical location, dietary habits, and oral hygiene practices [34]. Meanwhile, increased consumption of carbohydrates, increased acid in the mouth from vomiting, and decreased salivary calcium and phosphate could also increase the risk of dental caries during pregnancy [18]. During our analysis, we incorporated commonly known confounding factors, including demographic, socioeconomic, medical, and oral hygiene practices. The pregnant women enrolled in our study were all in their third trimester. During this specific time window, the variation in salivary flow rate and composition was generally minimal. We recognize that the lack of information on saliva features and dietary habits is a study limitation. Future studies, including assessments of these factors, are warranted to elucidate the relationship between salivary hormone levels and dental caries.

To further understand the role of each individual hormone and its relationship with the cariogenic pathogen, salivary *S. mutans* >10^5^ CFU/mL, which was acknowledged as an indicator for high caries risk, was selected to be a dependent variable to reflect cariogenic pathogen carriage in saliva and clinical caries risk status. Steroid hormones progesterone, estradiol, testosterone, and cortisol were highlighted to be positively associated with the increased risk of dental caries with significance (*p* < 0.05), while no significant association was found between dental caries status and thyroid hormones. This result indicates the potential direct or indirect relationship between steroid hormones and cariogenic pathogens. Previous studies have shown oral microbiome shifts during pregnancy with an increased proportion of anaerobic and aerobic bacteria, such as *Bacteroides melaninogenicus*, *Bifidobacterium dentium*, *Prevotella intermedia*, and *Porphyromonas gingivalis* [35], in which hormonal and immunologic changes were considered a critical role [11]. Studies have shown that increased levels of salivary progesterone during pregnancy are directly associated with increased levels of *Bifidobacteriaceae*, *Streptococcae*, and *Carnobacteriaceae* at the family level [12]. Meanwhile, the potential relationship between the increased level of *Candia* spp. in the oral cavity and late pregnancy has also been reported before [13]. Our results demonstrated increased salivary *C. albicans* levels in high-level hormonal clusters, while no significance was found in the covariate analysis. Although the mechanism remains unclear, the positive association between steroid hormones and the high levels of cariogenic pathogens could be explained by the host’s immune changes and the direct impact of hormones on oral microbial composition. Elevated estrogen and progesterone can inhibit innate and adaptive immunity at the systemic or local level, thereby facilitating the inflammatory state of the oral environment and opportunistic infection [8]. Meanwhile, some vulvovaginal studies have reported that estrogen and progesterone not only stimulate the production of glycogen from vaginal epithelium, which serves as a nutritional source for *Candida* growth [9] but also directly regulate the pathogenic features of *Candida* through the presence of biting proteins [25].

Samples in this study were collected during the daytime, between 9 am and 4 pm. Previous studies have reported that estriol and thyroid-stimulating hormone (TSH) in saliva remain relatively stable during daylight hours [36,37]. Conversely, cortisol concentrations typically rise after awakening, reaching peak levels around 30 min after awakening, and then continuously decline throughout the day [38]. Given the information above, we considered daytime collection to be an optimal and convenient collection time window for sex hormones and thyroid hormones. However, due to the fluctuating nature of cortisol, the cortisol result in this study should be interpreted with caution. Another limitation in analyzing factors associated with hormone levels in saliva is the exclusion of body mass index (BMI) from our study. It could be justified by the following: (1) the potential effect of BMI on salivary hormone levels is unclear and unconfirmed with limited available data [39], (2) for most steroid hormones, BMI adds very little to the variance accounted for by age in the regression model [40]; still, future studies are encouraged to include BMI into the covariate analysis to better understand potential factors related to salivary hormone levels during pregnancy.

In conclusion, the strength of this study, to our knowledge, is that it is the first to use the clustering method to analyze salivary hormones among socioeconomically disadvantaged pregnant women, and to evaluate the relationship between salivary hormones, dental caries conditions, and cariogenic pathogens. Conversely, the limitations of this study should be considered. Firstly, due to the nature of the cross-sectional study, we are not able to determine the causal relationship between hormone levels and the status of dental caries and cariogenic microbes. Meanwhile, to further elucidate the relationship between salivary hormones and clinical caries conditions, confounding factors, including dietary habits, salivary features, and diurnal changes in cortisol, need to be obtained and analyzed in future studies. Moreover, the study participants resided in Upstate New York and were all from underserved communities; the study results cannot be generalized to other populations or those residing outside of upstate New York. Longitudinal studies involving diverse populations and geographic locations are warranted in the future to further understand the role of hormones in oral microbial shifts and oral health during pregnancy.

## 5. Conclusions

This study demonstrated the feasibility of detecting salivary hormones during pregnancy. The study results indicated that salivary hormones, especially progesterone and estradiol, were significantly associated with a higher cariogenic risk among pregnant women. Moreover, our results indicated that salivary steroid hormones could serve as a potential indicator for caries risk evaluation beyond pregnant women.

## Figures and Tables

**Table 1:** Hormone detection rates and levels in saliva during the 3rd trimester (n = 181).

	Detection Rate	Mean (pg/mL) ± SD	Median (pg/mL)	Range (pg/mL)
Estradiol	92%	856.9 ± 1122.9	570.0	60.0–9770.0
Progesterone	97%	14,435.3 ± 15,182.6	10,100.0	230.0–97,430.0
Testosterone	77%	1131.6 ± 1777.0	590.0	10.0–15,100.0
Cortisol	99%	7252.7 ± 7402.1	5730.0	270.0–77,080.0
T3	71%	1105.0 ± 1989.3	380.0	10.0–17,040.0
T4	50%	686.3 ± 1258.0	30.0	30.0–9950.0

**Table 2:** Demographic–medical–oral characteristics of three hormonal clusters.

Categories		Total	Hormonal Clusters			
		n = 181	Low (n = 48)	Intermediate (n = 56)	High (n = 77)	*p* Value
***Demographic characteristics***						
Age (year)		27.6 ± 5.5	27.6 ± 5.7	28.0 ± 5.7	27.4 ± 5.3	0.83
Gestational weeks		33.6 ± 5.2	32.1 ± 3.6	34.9 ± 7.7	33.4 ± 3.1	0.31
Race	White	29.3%	41.7%	33.9%	18.2%	0.01
	Black	53.0%	41.7%	50%	62.3%	
	Other	17.7%	16.7%	16.1%	19.5%	
***Medical conditions***						
Diabetes		6.6%	6.3%	7.1%	6.5%	0.98
Hypertension		13.8%	14.6%	12.5%	14.3%	0.94
Asthma		12.7%	10.4%	16.1%	17.7%	0.65
Emotional conditions		33.7%	37.5%	37.5%	28.6%	0.45
Smoking		14.9%	4.2%	17.9%	19.5%	0.05
***Socioeconomic characteristics***						
Education	≤High school	58.0%	67.0%	64.9%	88%	0.17
	Associate degree	13.8%	4.2%	22.9%	25.0%	
	≥College	28.2%	29.2%	25.0%	29.9%	
Employment		50.8%	54.2%	51.8%	48.1%	0.79
Married		22.1%	27.1%	19.6%	20.8%	0.62
***Oral health***						
Tooth brushing	Twice/daily	65.2%	75%	62.5%	61.0%	0.25
	≤Once/daily	34.8%	25%	37.5	39.0%	
PI		1.6 ± 0.6	1.5 ± 0.6	1.8 ± 0.5	1.7 ± 0.6	0.11
DT		2.7 ± 3.7	1.8 ± 2.8	2.6 ± 2.7	3.4 ± 4.6	0.02
DS		4.1 ± 7.5	2.7 ± 4.6	3.5 ± 4.1	5.5 ± 10.2	0.03
ICDAS		3.5 ± 2.5	2.7 ± 2.3	3.4 ± 2.5	3.9 ± 2.5	<0.01
Salivary *S. mutans* Ln (CFU/mL × 10^5^)		11.5 ± 4.0	9.9 ± 5.5	12.5 ± 2.9	11.9 ± 3.2	<0.01
Salivary *C. albicans* Ln (CFU/mL × 10^2^)		3.2 ± 3.7	2.0 ± 2.99	3.5 ± 3.9	3.8 ± 3.7	0.02

DT: decayed teeth. DS: decayed surface. ICDAS: International Caries Detection and Assessment System.

**Table 3:** Factors associated with the pattern of salivary hormone clusters during pregnancy.

	Variables	Odds Ratio	Standard Error	Statistic	*p* Value	95%	
						Lower	Upper
Intermediate vs. Low	Intercept	0.34	1.46	−0.74	0.46	−3.95	1.79
	Age (year)	0.96	0.05	−0.84	0.4	−0.13	0.05
	Race (Black vs Non-Black)	2.77	0.42	2.40	0.02	0.19	1.85
	Married	1.31	0.52	0.52	0.61	−0.74	1.28
	Smoking	1.22	0.10	1.89	0.06	−0.01	0.40
	Hypertension	1.03	0.07	0.38	0.70	−0.12	0.17
	Emotional condition	1.00	0.39	−0.01	0.99	−0.76	0.76
	PI	10.01	0.86	2.68	0.01	0.62	3.99
	DT	0.53	0.45	−1.42	0.16	−1.51	0.24
	ICDAS	0.83	0.58	−0.32	0.75	−1.32	0.95
	*C. albicans* Ln (CFU/mL × 10^5^)	1.11	0.07	1.54	0.12	−0.03	0.24
	*S. mutans* Ln (CFU/mL × 10^5^)	1.09	0.05	1.76	0.08	−0.01	0.19
High vs. Low	Intercept	0.03	1.59	−2.29	0.02	−6.76	−0.53
	Age (year)	1.02	0.05	0.37	0.71	−0.07	0.11
	Race (Black vs. Non-Black)	1.63	0.44	1.13	0.26	−0.36	1.35
	Married	1.00	0.54	0.01	0.99	−1.06	1.06
	Smoking	1.03	0.11	0.28	0.78	−0.18	0.24
	Hypertension	0.97	0.08	−0.34	0.73	−0.18	0.13
	Emotional condition	1.83	0.41	1.49	0.14	−0.19	1.40
	PI	6.84	0.88	2.19	0.03	0.21	3.64
	DT	0.78	0.45	−0.54	0.59	−1.14	0.64
	ICDAS	0.85	0.62	−0.25	0.80	−1.37	1.06
	*C. albicans* Ln (CFU/mL×10^5^)	1.09	0.07	1.19	0.23	−0.06	0.23
	*S. mutans* Ln (CFU/mL×10^5^)	1.17	0.06	2.50	0.01	0.03	0.28
High vs. Intermediate	Intercept	12.95	1.42	1.80	0.07	−0.22	5.35
	Age (year)	0.95	0.04	−1.38	0.17	−0.13	0.02
	Race (Black vs. Non-Black)	1.69	0.38	1.39	0.17	−0.22	1.27
	Married	1.30	0.48	0.54	0.59	−0.68	1.21
	Smoking	1.18	0.09	1.75	0.08	−0.02	0.35
	Hypertension	1.06	0.06	0.98	0.33	−0.05	0.16
	Emotional condition	0.54	0.35	−1.72	0.09	−1.30	0.09
	PI	1.46	0.51	0.75	0.45	−0.62	1.38
	DT	0.68	0.40	−0.98	0.33	−1.16	0.39
	ICDAS	0.97	0.54	−0.05	0.96	−1.09	1.03
	*C. albicans* Ln (CFU/mL × 10^5^)	1.02	0.05	0.40	0.69	−0.08	0.13
	*S. mutans* Ln (CFU/mL × 10^5^)	0.93	0.06	−1.09	0.28	−0.19	0.05

**Table 4:** Salivary hormone levels in pregnant women with salivary *S. mutans* ≥10^5^ CFU/mL.

Hormones	Salivary *S. mutans* ≥10^5^ CFU/mL (n = 125)	Salivary *S. mutans* <10^5^ CFU/mL (n = 56)	*p* Value
Estradiol (pg/mL)	922.4 ± 1092.9	712.5 ± 1193.6	0.003
Progesterone (pg/mL)	16,315.2 ± 15,162.6	10,353.4 ± 14,661.0	<0.001
Testosterone (pg/mL)	1161.6 ± 1524.4	1067.3 ± 2265.2	0.01
Cortisol (pg/mL)	7432.9 ± 5459.1	6887.5 ± 10,601.5	0.09
T3 (pg/mL)	1032.3 ± 1626.4	1268.6 ± 2645.1	0.15
T4 (pg/mL)	640.7 ± 1079.1	797.0 ± 1597.6	0.86

## Data Availability

All data generated or analyzed during this study are included in this article. Further inquiries can be directed to the corresponding author.
